# Extracellular Vesicles From the Dermatophyte *Trichophyton interdigitale* Modulate Macrophage and Keratinocyte Functions

**DOI:** 10.3389/fimmu.2018.02343

**Published:** 2018-10-09

**Authors:** Tamires Aparecida Bitencourt, Caroline Patini Rezende, Natália Renault Quaresemin, Pedro Moreno, Otavio Hatanaka, Antonio Rossi, Nilce Maria Martinez-Rossi, Fausto Almeida

**Affiliations:** ^1^Department of Genetics, Ribeirao Preto Medical School, University of São Paulo, Ribeirao Preto, Brazil; ^2^Department of Biochemistry and Immunology, Ribeirao Preto Medical School, University of São Paulo, Ribeirao Preto, Brazil

**Keywords:** *Trichophyton interdigitale*, extracellular vesicles, innate immunity, fungal infection, macrophages, keratinocytes, nanoparticle-tracking analysis

## Abstract

The release of biomolecules critically affects all pathogens and their establishment of diseases. For the export of several biomolecules in diverse species, the use of extracellular vesicles (EVs) is considered to represent an alternative transport mechanism, but no study to date has investigated EVs from dermatophytes. Here, we describe biologically active EVs from the dermatophyte *Trichophyton interdigitale*, a causative agent of mycoses worldwide. EV preparations from *T. interdigitale* were examined using nanoparticle-tracking analysis, which revealed vesicular structures 20–380 nm in diameter. These vesicles induced the production of proinflammatory mediators by bone marrow-derived macrophages (BMDMs) and keratinocytes in a dose-dependent manner, and an addition of the EVs to BMDMs also stimulated the transcription of the M1-polarization marker iNOS (inducible nitric oxide synthase) and diminished the expression of the M2 markers arginase-1 and Ym-1. The observed M1 macrophages' polarization triggered by EVs was abolished in cells obtained from knockout Toll-like receptor-2 mice. Also, the EVs-induced productions of pro-inflammatory mediators were blocked too. Furthermore, the EVs from *T. interdigitale* enhanced the fungicidal activity of BMDMs. These results suggest that EVs from *T. interdigitale* can modulate the innate immune response of the host and influence the interaction between *T. interdigitale* and host immune cells. Our findings thus open new areas of investigation into the host-parasite relationship in dermatophytosis.

## Introduction

Dermatophytosis is the most common type of superficial mycosis in humans (1). Over the past few years, increasing numbers of dermatophytosis cases have been linked to diabetes or aging or to patients being immunocompromised (2). *Trichophyton rubrum* and *Trichophyton interdigitale* have been described as the most common causative agents of mycoses worldwide (3, 4); the two fungi infect keratinized structures in the host, such as skin and nails, and cause dermatophytoses or tineas, which are commonly known as athlete's foot, onychomycosis, ringworm, and jock itch (3, 5–7). These infections are typically chronic and produce mild symptoms because of the adaptive mechanisms developed by the fungi to evade the host immune response (8).

All pathogens and their ability to establish diseases are critically affected by the secretion of diverse biomolecules (9, 10), and certain studies have shown that fungi release several virulence factors into the extracellular space by using extracellular vesicles (EVs) (11–15). These EVs transport numerous molecules and thereby contribute to fungal virulence and the modulation of the host immune-response profile (16–21).

EVs, which are produced by eukaryotes, bacteria, and archaea (22), are spherical, lipid-bilayered membrane structures that range in diameter from 20 to 500 nm. The EV cargo includes proteins, nucleic acids, lipids, pigments, polysaccharides, and toxins, and thus these vesicles play essential roles in microbial structure and pathogenesis (10, 15). Fungal EVs were first isolated and characterized in 2007 from the pathogenic fungus *Cryptococcus neoformans* (11), and, since then, the research on fungal EVs has increased considerably and EVs have been purified and characterized from culture supernatants in numerous studies (12–14, 17, 23). These studies characterizing EVs and their immunomodulatory activity contribute toward our understanding of the role of EVs in host-pathogen interactions. However, no study to date has focused on isolating and describing EVs from dermatophytes.

Here, we report for the first time that a dermatophyte produces EVs. We found that *T. interdigitale* culture supernatants contained vesicles, and by using various methods, we demonstrated that EVs from *T. interdigitale* were biologically active; the EVs induced macrophages and keratinocytes to release proinflammatory mediators and enhanced the fungicidal ability of macrophages, which suggests that these EVs play a role in fungal pathogenesis. Our findings demonstrate a previously unrecognized phenomenon in dermatophytes—EV production—that is relevant to aspects such as microbial physiology and pathogenesis, and further provide new insights into the mechanisms by which secreted molecules reach the extracellular environment and their potential to influence the interaction of *T. interdigitale* with host immune cells.

## Methods

### Ethics statement

All animal use complied with the standards described in the Ethical Principles Guide for the Care and Use of Laboratory Animals adopted by the Brazilian College of Animal Experimentation. This study was approved by the Committee of Ethics in Animal Research of the Ribeirao Preto Medical School at the University of Sao Paulo (RPMS-USP; protocol 2.101.529).

### Mice and *T. interdigitale* isolates

We used 8-12-week-old male C57BL/6 (wild-type, WT), TLR2 Knockout (TLR2^−/−^), and TLR4 Knockout (TLR4^−/−^) mice. The animals were housed in the animal facility of the RPMS-USP, under optimized hygienic conditions.

*T. interdigitale* experiments were conducted using the strain H6 (ATCC MYA3108) (4, 24); this clinical isolate was maintained by subcultivation in malt-extract agar [2% glucose, 2% malt extract, 0.1% peptone (w/v), pH 5.7] for 21 days at 28°C. We prepared suspensions of conidia from 21-day-old culture plates that were flooded with sterile 0.9% NaCl, and then filtered the suspensions through fiberglass to remove mycelium debris. The concentration of conidia in the filtrate was estimated using a Neubauer chamber.

### EV isolation

EVs were isolated as previously described for *C. neoformans* (11), with slight modifications. We cultivated 10^6^ conidia in minimal medium (25) containing dextrose [55 mM] and NaNO_3_ (70 mM) for 3 days at 28°C with continuous shaking, and then sequentially centrifuged the culture medium at 6,000 × *g* and 15,000 × *g* to obtain separated supernatants. The pellets were discarded and the supernatants were concentrated using a 100-kDa-cutoff Amicon ultrafiltration system (Millipore, Billerica, MA, USA), and the obtained material was ultracentrifuged at 100,000 × *g* for 1 h at 4°C. The supernatants were discarded, and the pellets were washed thrice with PBS by repeatedly resuspending and centrifuging them at 100,000 × *g* for 1 h at 4°C.

### Nanoparticle-tracking analysis (NTA)

Size-distribution analysis and quantification of EV preparations were performed on a NanoSight NS300 (Malvern Instruments, Malvern, UK) equipped with fast video capture and particle-tracking software. Purified vesicles from *T. interdigitale* were diluted into 1 mL of PBS and disaggregated using a syringe and needle (1-mL 29-gauge × ½), and each sample was then injected into a NanoSight sample cubicle. Both scatter and fluorescence-capture settings (such as focus, camera, and gain settings) were optimized to make particle tracks visible, and then measurements were obtained in triplicate and analyzed using NanoSight software (version 3.2.16). The data on the sizes of EVs from *T. interdigitale* are expressed as the calculated means ± SD of size distribution.

### Preparation of BMDMs and keratinocytes

BMDMs were generated as previously described (26), with slight modifications (27). Bone marrow cells were collected from the femurs and tibias of 8–12-week-old (adult) C57BL/6 (wild-type, WT), TLR2 Knockout (TLR2^−/−^), and TLR4 Knockout (TLR4^−/−^) mice by flushing with RPMI 1640 medium to release the cells, which were then cultured for 6 days in RPMI 1640 medium supplemented with 20% fetal cow serum and 30% L-929 cell-conditioned medium. Non-adherent cells were removed, and the adherent cells (majority macrophages) were collected and washed twice with cold PBS. Cell concentrations were determined using a Neubauer chamber, and the cells were plated in RPMI 1640 medium containing 10% fetal bovine serum (FBS) and 5% L-929 cell-conditioned medium and used for cytokine-detection assays (1.5 × 10^6^/mL; 7.5 × 10^5^ cells/well; 48-well plates) or quantitative reverse-transcription-PCR (qRT-PCR) analysis [2 × 10^6^/mL; 1 × 10^6^ cells/well; 24-well plates].

For the keratinocyte experiments, we used the human keratinocyte cell line HaCaT; the cells were cultured in Dulbecco's modified Eagle medium (DMEM; Gibco, New York, NY, USA) supplemented with 10% FBS and penicillin-streptomycin (100 U/mL) at 37°C in a humidified 5% CO_2_ balanced air incubator. HaCaT cells were plated at 3 × 10^5^ cells/well in 6-well plates, and 24 h later, the medium was changed to serum-free DMEM containing EVs at various concentrations.

BMDMs and HaCaT cells were incubated with different concentrations of EVs (10^3^-10^7^ particles/mL), lipopolysaccharide (LPS; 1 μg/mL) plus interferon (IFN)-γ (2 ng/mL), interleukin (IL)-4 (50 ng/mL) plus IL-10 (50 ng/mL), or the medium alone. The BMDMs were cultured for 6 h for qRT-PCR analysis, 48 h for determining the levels of tumor necrosis factor (TNF)-α, IL-6, IL-1β, and nitric oxide (NO), and 4 and 48 h for quantifying their phagocytosis and killing of *T. interdigitale*, respectively. The HaCaT cells were cultured for 24 h and then used for measuring TNF-α, IL-6, IL-1β, IL-8, and NO levels.

### Determination of NO production

The amount of NO present in BMDM and keratinocyte culture supernatants was quantified by analyzing the accumulation of nitrite in the monolayer supernatants by using the standard Griess reaction (28). Briefly, 50 mL of supernatant was incubated with an equal volume of Griess reagent (1% sulfanilamide, 0.1% naphthyl ethylenediamine dihydrochloride, 2.5% H_3_PO_4_) for 10 min at room temperature, and then the 550-nm absorbance was measured using a microplate-scanning spectrophotometer (Power Wave-X; BioTek Instruments, Inc., Winooski, VT, USA). The measured absorbance was converted into micromolar concentrations of NO based on a standard curve generated using a known concentration of NaNO_2_ diluted in RPMI 1640 medium.

### Cytokine measurement

Supernatants of stimulated BMDMs and HaCaT cells were used to quantify the levels of TNF-α, IL-6, IL-1β, and IL-8. The cytokines were measured using capture enzyme-linked immunosorbent assay (ELISA) performed with antibody pairs purchased from BD Biosciences (Pharmingen, San Diego, CA, USA); the ELISA was performed according to the manufacturer's protocol, and the concentrations were calculated from standard curves generated by a curve-fitting program. The absorbance was read at 450 nm in a microplate-scanning spectrophotometer (Power Wave-X).

### qRT-PCR analysis

qRT-PCR was performed as previously described (18), with slight modifications. BMDMs (2 × 10^6^/mL; 1 × 10^6^ cells/well; 24-well plates) were stimulated for 6 h and then total RNA was extracted from the cells by using Trizol Reagent (Invitrogen, Life Technologies, Camarillo, CA, USA) according to the manufacturer's protocol. The RNA was reverse-transcribed into cDNA by using an ImProm-II Reverse Transcription System (Promega, Fitchburg, WI, USA) with oligo(dT). The qRT-PCR was performed in 15-μl reactions containing SsoFast™ EvaGreen (Bio-Rad Laboratories, Hercules, CA, USA), and a Bio-Rad CFX96 Real-Time PCR System (Bio-Rad Laboratories) was used for monitoring the reactions, which were under these conditions: 50°C for 2 min, 95°C for 10 min, and 40 cycles of 95°C for 15 s and 60°C for 1 min. All transcript levels were quantified using the ΔΔCt method and normalized relative to β-actin expression. The PCR primers used were the following: β-actin, F: CCTAAGGCCAACCGTGAAAA / R: GAGGCATACAGGGACAGCACA; inducible nitric oxide synthase (iNOS), F: CCGAAGCAAACATCACATTCA / R: GGTCTAAAGGCTCCGGGCT; Ym-1, F: TCACAGGTCTGGCAATTCTTCTG / R: ACTCCCTTCTATTGGCCTGTCC; and arginase-1, F: GTTCCCAGATGTACCAGGATTC / R: CGATGTCTTTGGCAGATATGC.

### Assays of fungus phagocytosis and killing by macrophages

For the phagocytosis assay, BMDMs were seeded at 2 × 10^5^ cells/well on 13-mm glass coverslips placed in 24-well plates and cultured at 37°C and 5% CO_2_ in DMEM supplemented with 10% FBS; before adding *T. interdigitale*, the cells were treated with EVs (10^7^ particles/mL), INF-γ (50 ng/mL), or the medium alone. The macrophages were challenged with 2 × 10^5^ conidia from *T. interdigitale* (macrophages:conidia = 1:1) in the 24-well plates for 4 h at 37°C, and then the glass coverslips were washed with PBS and stained with Giemsa. An average of 100 macrophages were counted when determining the percentage of macrophages that had internalized at least one conidium (P) and the average number of fungal cells in these macrophages (F). The phagocytic index (I) was calculated as P × F (29).

The phagocytic cells obtained as described above, with slight modifications, were used to evaluate the killing of conidia: 5 × 10^5^ conidia from *T. interdigitale* (macrophages:conidia = 1:1) were incubated at 37°C and 5% CO_2_ for 48 h in DMEM supplemented with 10% FBS, and before adding *T. interdigitale* to BMDMs, the cells were treated under the same conditions as those described above for the phagocytosis assay. The 24-well microplates were centrifuged for 10 min at 3,500 rpm, the culture supernatants were discarded, and the cells were washed with PBS to remove any fungal cells that had not been ingested. The BMDMs were lysed with cold water, and the lysates were plated on potato dextrose agar medium and incubated at 28°C for 72 h. The samples were analyzed for the presence of viable fungal cells by determining the colony forming units (CFU).

### Statistical analysis

Data shown are either the means of or representative results from at least 3 independent experiments, each performed in triplicate. All statistical analyses and comparisons were performed using *GraphPad Prism* Software version 6.0 (GraphPad Software, San Diego, CA, USA). One-way ANOVA and Turkey's multiple comparison posttests were applied. *P* < 0.05 was considered statistically significant.

### Data availability

All relevant data are contained within the manuscript.

## Results

### EV production by *T. interdigitale*

**Figure 1:**
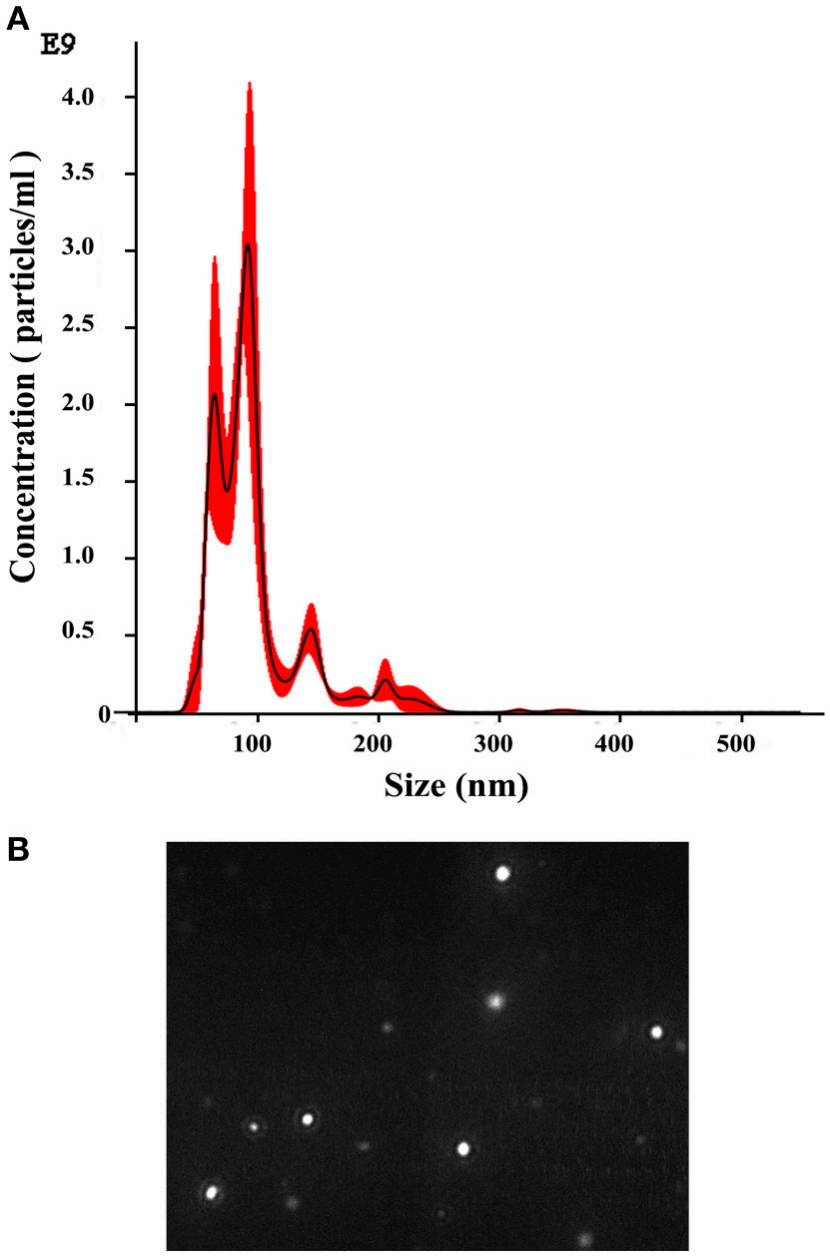
Nanoparticle-tracking analysis of extracellular vesicles (EVs) produced by *T. interdigitale*. EVs were purified from *T. interdigitale* culture supernatants and quantified using a NanoSight NS300. **(A)** Histogram showing the EV particle-size distribution (EVs × 10^9^/mL vs size in nanometers). **(B)** Screenshot from video recorded using the NanoSight NS300, showing the distribution of EVs from *T. interdigitale*.

The production of EVs by several fungi and other microbes has been described; thus, we sought to determine whether dermatophytes also produce EVs, and we performed experiments to detect vesicles in *T. interdigitale* culture supernatants. To isolate EVs from culture supernatants, 1 × 10^6^ conidia were cultivated for 72 h in minimal medium, and the collected culture supernatants were dialyzed and ultracentrifuged. Subsequently, the results of NTA showed that EVs were produced by *T. interdigitale* (Figure 1). Vesicles ranging in diameter from 20 to 380 nm were detected, and although the vesicle size varied substantially, the mean/mode diameter of most vesicles was approximately 110 nm (Figure 1A). The size distribution and screening profile of these EVs are shown in screenshot of video recordings from the NanoSight NS300 system (Figure 1B). To confirm that the vesicles originated from live cells and not from membranes released from dead cells, we analyzed a control preparation obtained from culture supernatants of *T. interdigitale* conidia that had been killed by heating at 90°C for 2 h and then inoculated in minimal medium and cultivated in parallel under the same conditions as those used for the live cells. Our NTA results demonstrated that no nanoparticles were generated from the heat-killed *T. interdigitale* conidia (data not shown). Similar results have previously been obtained for *C. neoformans* (11) and *Paracoccidioides brasiliensis* (14). These findings confirm that exosomes are produced by *T. interdigitale*.

### *T. interdigitale* EVs induce a proinflammatory profile in BMDMs

**Figure 2:**
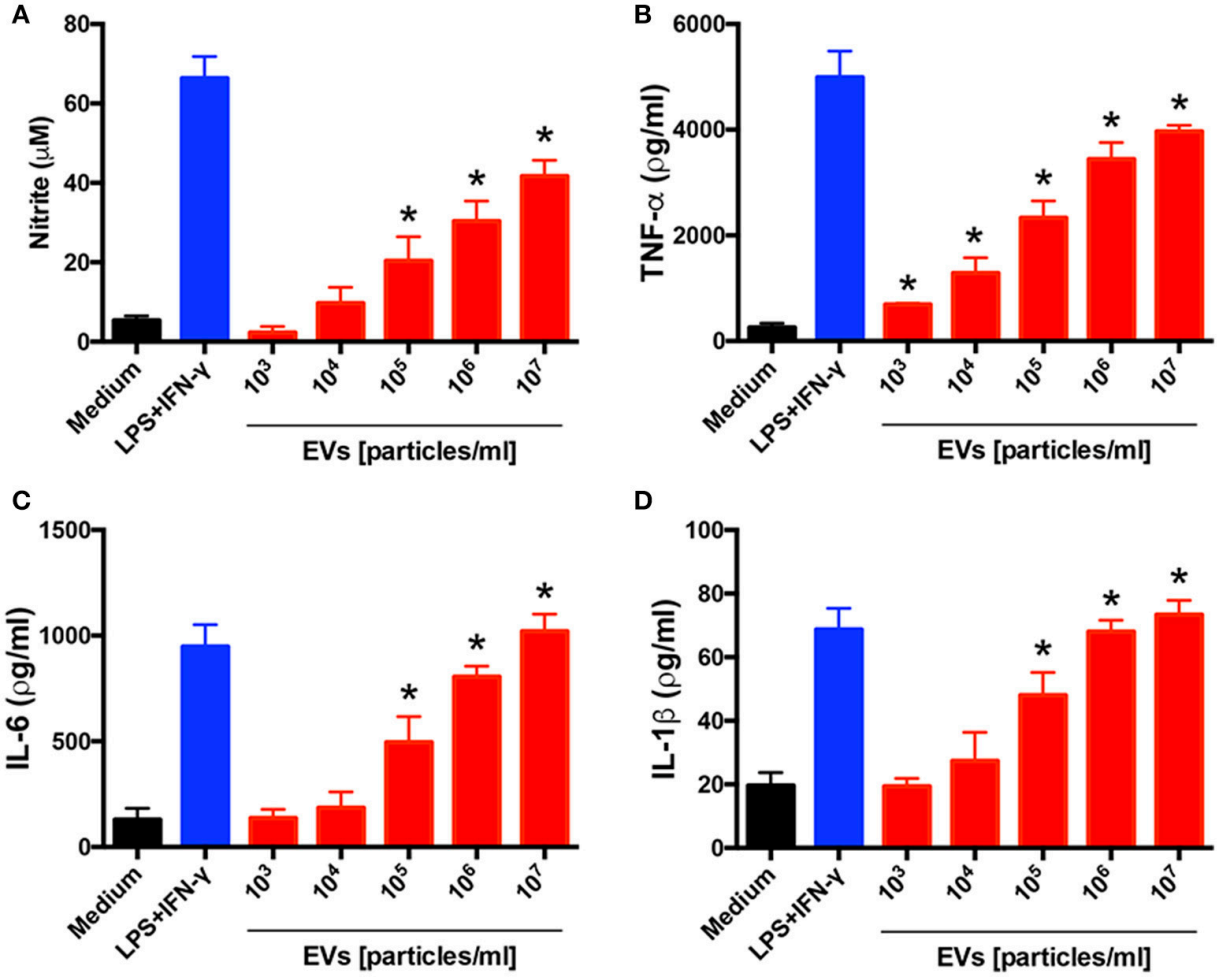
Extracellular vesicles (EVs) from *T. interdigitale* induce the production of proinflammatory mediators by bone marrow-derived macrophages (BMDMs). BMDMs from C57BL/6 mice were incubated at 37°C for 48 h with the indicated amounts of EVs isolated from *T. interdigitale* (x-axis). The medium and LPS (1 μg/mL) plus IFN-γ (2 ng/mL) were used as negative and positive controls, respectively. The culture supernatants were assessed for the concentrations of nitrite **(A)**, TNF-α **(B)**, IL-6 **(C)**, and IL-1β **(D)**. Data are representative of 3 experiments. The results are expressed as means ± SEM and are shown relative to the levels in non-stimulated cells (medium only). *P < 0.05.

To investigate the influence of EVs on the host immune-response profile, we tested whether the EVs from *T. interdigitale* stimulated BMDMs: We added different amounts of EVs to BMDMs and assessed the culture supernatants for the production of cytokines and NO after incubation for 48 h. EVs from *T. interdigitale* stimulated the release of NO, TNF-α, IL-6, and IL-1β in a dose-dependent manner (Figure 2). However, IL-10 levels in the treated cells were as low as the levels in non-stimulated BMDMs (data not shown). These results suggest that EVs induce BMDMs to produce proinflammatory mediators.

### *T. interdigitale* EVs induce a proinflammatory profile in keratinocytes

**Figure 3:**
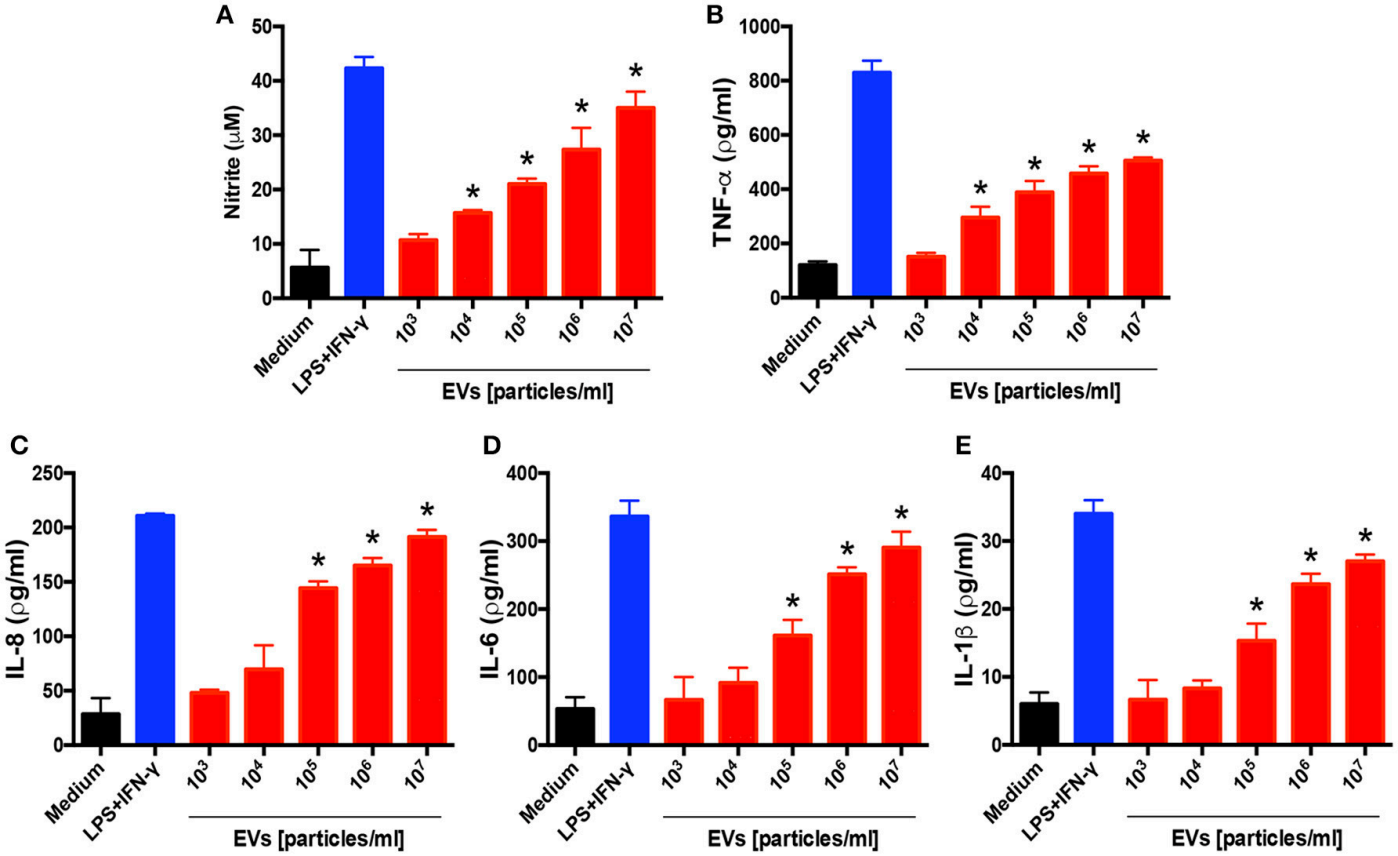
Extracellular vesicles (EVs) from *T. interdigitale* induce the production of proinflammatory mediators by keratinocytes. Human keratinocyte cell line HaCaT cells were incubated at 37°C for 24 h with the indicated amounts of EVs from *T. interdigitale* (x-axis). The medium and LPS (1 μg/mL) plus IFN-γ (2 ng/mL) were used as negative and positive controls, respectively. The culture supernatants were assessed for the concentrations of nitrite **(A)**, TNF-α **(B)**, IL-8 **(C)**, IL-6 **(D)**, and IL-1β **(E)**. Data are representative of 3 experiments. The results are expressed as means ± SEM and are shown relative to the levels in non-stimulated cells (medium only). *P < 0.05.

We also evaluated the influence of EVs on a human keratinocyte cell line: HaCaT cells were incubated for 48 h with different amounts of EVs, and the culture supernatants were assessed for cytokine and NO production. The levels of NO, TNF-α, IL-6, IL-1β, and IL-8 were markedly increased in response to the EV stimulus in a dose-dependent manner (Figure 3). These findings agree with the results showing that the EVs induced a proinflammatory profile in BMDMs.

### *T. interdigitale* EVs induce the macrophage M1 phenotype

**Figure 4:**
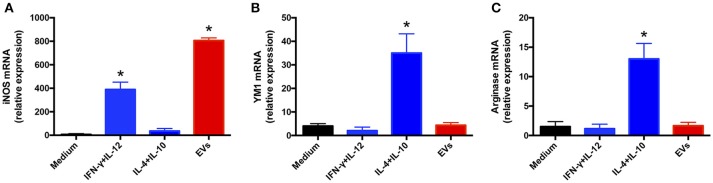
Extracellular vesicles (EVs) from *T. interdigitale* promote classical macrophage activation. Bone marrow-derived macrophages (BMDMs) from C57BL/6 mice were incubated at 37°C for 6 h with EVs (10^7^ particles/mL and with either IFN-γ (2 ng/mL) plus IL-12p40 (50 ng/mL) as M1 inducers (classical activation) or IL-10 plus IL-4 (both at 50 ng/mL) as M2 inducers (alternative activation). The medium was used as the negative control. RNA was extracted and converted into cDNA and real-time PCR was used to evaluate the relative expression of iNOS (**A**), Ym-1 (**B**), and arginase-1 (**C**). Data are representative of 3 experiments. The results are expressed as means ± SEM and are compared to the results obtained for the negative control. *P < 0.05.

EV induction of a proinflammatory profile in BMDMs and keratinocytes suggested that the EVs favored the development of the “classical” M1 activation phenotype in macrophages. To test this possibility, we extracted total RNA from BMDMs incubated with 10^7^ EVs for 6 h and performed relative quantification of the transcripts of M1 (iNOS) and M2 (arginase-1 and Ym-1) polarization markers. The iNOS mRNA level was increased 800-fold in EV-stimulated BMDMs, a response higher than that induced by IFN-γ plus IL-12 (Figure 4A). By contrast, the mRNA levels of Ym-1 (Figure 4B) and arginase-1 (Figure 4C) in the presence of vesicles remained close to that measured in non-stimulated cells. These results suggest that *T. interdigitale* EVs promote BMDM polarization toward the “classical” M1 phenotype.

### TLR2 is crucial for the macrophage release of proinflammatory cytokines induced by *T. interdigitale* EVs

**Figure 5:**
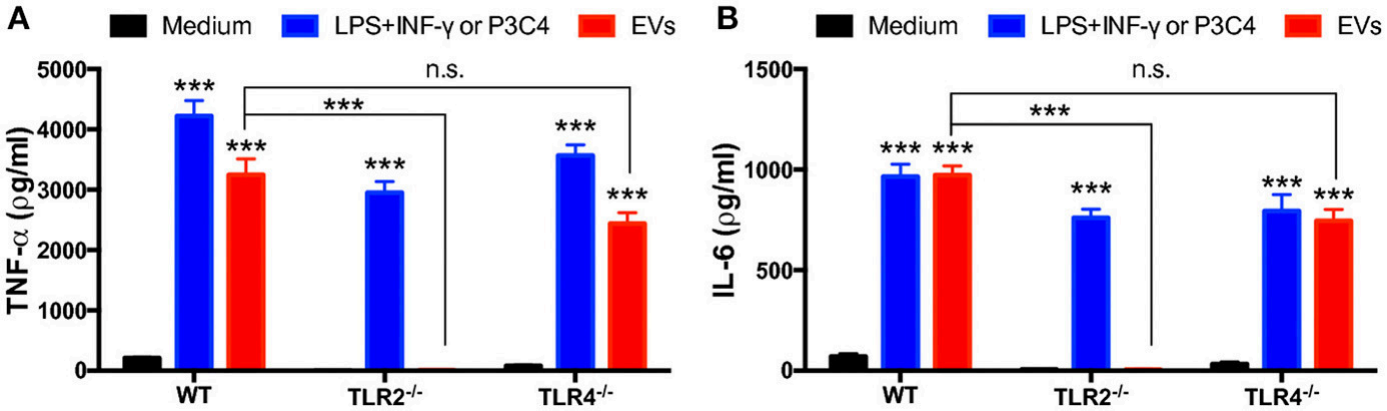
Involvement of TLR2 and TLR4 in the proinflammatory cytokine production induced by *T. interdigitale* EVs. Bone marrow-derived macrophages (BMDMs) obtained from WT, TLR2^−/−^, and TLR4^−/−^ mice were incubated at 37°C for 48 h with EVs (10^7^ particles/mL). A mixture of LPS (1 μg/mL) plus IFN-γ (2 ng/mL) were used as positive controls for WT and TLR2^−/−^ macrophages; Pam3CSK4 (100 ng/mL) was used as the positive control for TLR4^−/−^ macrophages. The cultures supernatants were assessed for the concentration of TNF-α (**A**) and IL-6 (**B**). Data are representative of 3 experiments. The results are expressed as means ± SEM and are shown relative to the levels in non-stimulated cells (medium only). ***P < 0.001, and non-significant differences (n.s.).

Given that *Trichophyton rubrum* interacts with TLR2 and TLR4 on antigen-presenting cells and promotes cytokines production (30), we evaluated the relevance of TLR2 and TLR4 in the production of inflammatory mediators induced by *T. interdigitale* EVs. We stimulated BMDMs obtained from WT, TLR2^−/−^, or TLR4^−/−^ mice with *T. interdigitale* EVs for 48 h and quantified the levels of TNF-α and IL-6 in the cell supernatants. We verified that the lack of TLR2 abolished the EVs-induced production of TNF-α and IL-6 (Figures 5A,B, respectively). The absence of TLR4, in turn, did not affect the TNF-α and IL-6 production. We conclude that TLR2 participates in the induction of proinflammatory cytokine production in macrophages stimulated by *T. interdigitale* EVs.

### TLR2 is crucial for the classical polarization of macrophages induced by *T. interdigitale* EVs

**Figure 6:**
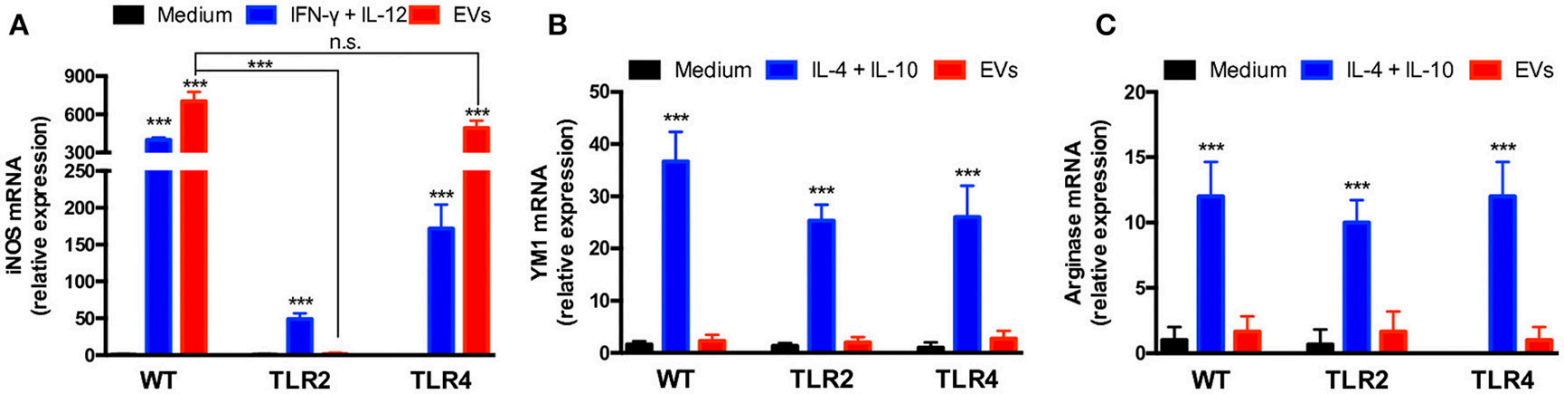
Involvement of TLR2 in the activation of bone marrow-derived macrophages (BMDMs) by *T. interdigitale* EVs. BMDMs from WT, TLR2^−/−^, and TLR4^−/−^ mice were stimulated for 6 h with EVs (10^7^ particles/mL). As positive control for the M1 activation, the mixture of INF-γ (2 ng/mL) plus IL-12p40 (50 ng/mL) was used. For M2 activation, a mixture of IL-10 plus IL-4 (50 ng/mL both) was used. The medium alone was used as negative control. RNA was extracted and converted into cDNA and real-time PCR was used to evaluate the relative expression of iNOS (**A**), Ym-1 (**B**), and arginase-1 (**C**). Data are representative of 3 experiments. The results are expressed as means ± SEM and are compared to the results obtained for the negative control. ***P < 0.001, and non-significant differences (n.s.).

To expand the investigation of the roles of TLR2 and TLR4 on the effects of *T. interdigitale* EVs on macrophages, we verified whether the M1 polarization of these cells promoted by *T. interdigitale* EVs could be affected by the absence of TLR2 and TLR4. Following the stimulation of BMDMs from WT, TLR2^−/−^, or TLR4^−/−^ mice, the cells were assessed for the expression of iNOS, YM-1, and Arginase-1. The iNOs expression stimulated by *T. interdigitale* EVs was not affected by the absence of TLR4, but was blocked in TLR2 BMDMs (Figure 6A). The expression of M2 polarization markers (YM-1 and Arginase-1), which did not respond to the *T. interdigitale* EVs stimulus, remained in cells from TLR2^−/−^ and TLR4^−/−^ mice at levels as low as those verified in non-stimulated macrophages (medium; Figures 6B,C). Therefore, we conclude that TLR2 is critical for the *T. interdigitale* EVs-induced M1 polarization of macrophages.

### EVs produced by *T. interdigitale* stimulate fungicidal activity of macrophages

**Figure 7:**
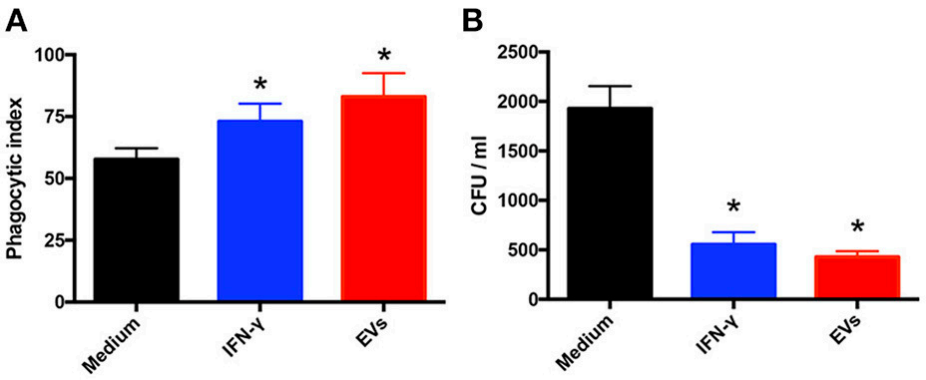
Extracellular vesicles (EVs) from *T. interdigitale* enhance fungicidal activity of macrophages. (**A**) Before the addition of *T. interdigitale* to bone marrow-derived macrophages (BMDMs; conidia:macrophages = 1:1), the cells were treated for 30 min with EVs (10^7^ particles/mL), IFN-γ (50 ng/mL), or the medium and then plated on glass coverslips; after incubation for 4 h at 37°C, the coverslips were washed with PBS, and the cells were stained with Giemsa and the phagocytic index determined. (**B**) Alternatively, BMDMs were infected with *T. interdigitale* (macrophages:conidia = 1:1) for 48 h and then the cells were washed and lysed for the detection of viable intracellular fungal cells based on measuring their CFU. Data are representative of 3 experiments. The results are expressed as means ± SEM and are compared to the results obtained for the negative control. *P < 0.05.

Lastly, because EV stimulation of macrophages and keratinocytes induced the production of proinflammatory mediators, we ascertained whether EVs promote *T. interdigitale* phagocytosis and killing by macrophages. First, we determined the phagocytic index of BMDMs. The macrophages were stimulated with EVs from *T. interdigitale* for 30 min before the addition of *T. interdigitale* conidia, and then the cocultures were maintained for 4 h. The presence of EVs during the phagocytosis assay enhanced conidium engulfment by >25% (Figure 7A), and the phagocytic index calculated for IFN-γ- and EV-stimulated cells showed a similar increase relative to the phagocytic index for non-stimulated cells (Figure 7A). Furthermore, analyses performed on BMDM lysates after 48 h incubation showed that stimulation with either IFN-γ or EVs led to CFU numbers being lower than those measured with non-stimulated BMDMs (Figure 7B). These results indicate that EVs not only promote the uptake of *T. interdigitale* by macrophages, but also enhance the fungicidal activity of macrophages.

## Discussion

We have reported here for the first time that a dermatophyte produces EVs. We have described the production of EVs by *T. interdigitale* and the isolation of the EVs, and we have shown that *T. interdigitale* EVs induce the activation of macrophages and keratinocytes and that the EVs can modulate the innate immunity of the host. Furthermore, we have demonstrated that EVs from *T. interdigitale* can enhance the fungicidal capacity of macrophages.

Almost all living cells produce exosomes, which have been shown to play roles in cell communication, physiology, and pathogenesis (10, 15). These studies have clearly demonstrated that exosomes influence both microorganism pathogenesis and the host immune response. Although the relevant roles during infection have been attributed to EVs in these cases, our understanding of the functions of EVs in fungal infections remains rudimentary.

A notable finding in this study was that EV-stimulated BMDMs released proinflammatory mediators and that the phagocytic index of these cells was increased, which was in accord with the enhanced killing of *T. interdigitale*. This result agrees with the finding that BMDMs stimulated with EVs from *T. interdigitale* showed an increase in the levels of NO and proinflammatory mediators (such as TNF-α, IL-6, and IL-1β). Results similar to those presented here were also obtained with *P. brasiliensis* EVs, which favored the development of the Th1 immune response and enhanced the fungicidal activity of murine peritoneal macrophages (18). In the case of the yeast *C. neoformans*, EVs increased the levels of TNF-α, transforming growth factor-β, and IL-10 produced by RAW 264.7 murine macrophages and enhanced the capacity of these cells to kill *C. neoformans* (16). In a recent study, EVs from *Cryptococcus gattii* were shown to be taken up by infected macrophages, which indicated an essential role of the EVs in the fungal pathogenesis (31). Furthermore, *Candida albicans* EVs induced the production of NO, IL-12p40, IL-10, and TNF-α by BMDMs and dendritic cells (17), and treatment of macrophages and dendritic cells with these EVs upregulated the expression of CD86 and MCH-II (17). Thus, given that fungal EVs were shown to induce the production of cytokines and NO, these studies support the notion that fungal EVs play a decisive role in the induction of antifungal immunity.

The recognition and elimination of fungal pathogens depend of several cells of the innate immune system (32), and the importance of macrophages in combating fungal infections has been widely demonstrated. Moreover, the switching of the macrophage activation phenotype between M1 and M2 is known to be crucial for the effective control of several fungal pathogens (32). We demonstrated that BMDMs and keratinocytes released proinflammatory mediators in response to EV stimulation, which could contribute to the development of M1 macrophages. Notably, EVs from *T. interdigitale* upregulated the expression of iNOS but did not alter the expression of arginase-1 and Ym-1, which suggests polarization of the macrophages toward the “classical” M1 phenotype. Accordingly, EVs from *P. brasiliensis* were also found to promote the M1 polarization of macrophages (18). The mechanism behind the mentioned events typically involves TLRs. We found that TLR participates in cytokine production induced by *T. interdigitale* EVs on BMDMs and that TLR2 is required not only to induce the release of proinflammatory mediators but also to promote the M1 polarization of BMDMs in response to *T. interdigitale* EVs. However, there are few studies on the effects of TLRs activation during the course of this infection. Patients with *T. rubrum* showed reduced expression of TLR4 in localized and disseminated dermatophytosis, and no differences were verified in TLR2 expression (33). On the other hand, in another report, *T. rubrum* seems to manipulate the host defense, which the conidia were able to down-modulate TLRs (30). This observation partially supports our data regarding the participation of TLR2 in the response to the *T. interdigitale* EVs stimulus.

The role of certain immune-response systems (such as the Th1, Th2, and Th17 responses) in the immunity toward several fungal pathogens is well established. However, the functions of these responses in *T. rubrum*, and particularly in *T. interdigitale*, remain poorly understood (34). A recent study showed that neither the IL-17 response nor adaptive immunity was required for fungal control of *T. rubrum* (35). Moreover, *T. rubrum* was reported to induce IL-1β production by macrophages, which, in turn, played essential roles in the immune response against *T. rubrum* (36), and the culture supernatant of *T. rubrum* was further shown to activate the innate immune response of HaCaT cells (37). The findings of these studies agree with our results showing that keratinocytes stimulated with EVs from *T. interdigitale* showed increased production of NO and proinflammatory mediators.

Fungal EVs are recognized to carry proteins, toxins, nucleic acids, lipids, and several other molecules (10, 15). However, we did not verify here the presence of essential virulence factors in the EVs from *T. interdigitale*. Therefore, the specific cargo molecules carried by *T. interdigitale* EVs must be identified in future studies to clarify the potential functions of the EVs.

In this report, we have described EV production by *T. interdigitale* and have shown that these EVs can modulate *in vitro* the functions of macrophages and keratinocytes. Our findings provide new insights for future investigations into the dermatophytosis and suggest a new target for the design of therapeutic agents to combat this critical mycosis.

## Author contributions

All authors contributed to the research design and data analyses. TB, CR, PM, OH, NQ, and FA performed the experiments. AR, NM-R, and FA contributed reagents, materials, analysis tools. TB, CR, AR, NM-R, and FA Wrote the paper.

### Conflict of interest statement

The authors declare that the research was conducted in the absence of any commercial or financial relationships that could be construed as a potential conflict of interest. The reviewer CS declared a shared affiliation, with no collaboration, with several of the authors TB, CR, NQ, PM, OH, AR, NM-R and FA to the handling Editor.

## References

[1] Brown GD Denning DW Gow NAR Levitz SM Netea MG White TC. (2012). Hidden killers: human fungal infections. Sci Trans Med. 4:165rv13. 10.1126/scitranslmed.3004404 23253612

[2] Kaur R Panda PS Sardana K Khan S. Mycological pattern of dermatomycoses in a tertiary care hospital. J Trop Med. (2015) 2015:157828. 10.1155/2015/157828 26491453 PMC4605448

[3] Weitzman I Summerbell RC. The dermatophytes. Clin Microbiol Rev. (1995) 8:240–59. 7621400 10.1128/cmr.8.2.240 PMC172857

[4] Persinoti GF Martinez DA Li W Dogen A Billmyre RB Averette A. Whole-genome analysis illustrates global clonal population structure of the ubiquitous dermatophyte Pathogen *Trichophyton Rubrum*. Genetics (2018) 208:1657–69. 10.1534/genetics.117.300573 29467168 PMC5887155

[5] Havlickova B Czaika VA Friedrich M. Epidemiological trends in skin mycoses worldwide. Mycoses (2008) 51 (Suppl. 4):2–15. 10.1111/j.1439-0507.2008.01606.x 18783559

[6] Seebacher C Bouchara JP Mignon B. Updates on the epidemiology of dermatophyte infections. Mycopathologia (2008) 166:335–52. 10.1007/s11046-008-9100-9 18478365

[7] Nenoff P Kruger C Ginter-Hanselmayer G Tietz HJ Mycology - an update. Part 1: Dermatomycoses: causative agents, epidemiology and pathogenesis. J Dtsch Dermatol Ges. (2014) 12:188–209; quiz 210, 188-211; quiz 212. 10.1111/ddg.12245 24533779

[8] Hube B Hay R Brasch J Veraldi S Schaller M. Dermatomycoses and inflammation: the adaptive balance between growth, damage, and survival. J Mycol Med. (2015) 25:e44–58. 10.1016/j.mycmed.2014.11.002 25662199

[9] Almeida F Wolf JM Casadevall A. Virulence-associated enzymes of *Cryptococcus neoformans*. Eukaryot Cell (2015) 14:1173–85. 10.1128/EC.00103-15 26453651 PMC4664877

[10] Brown L Wolf JM Prados-Rosales R Casadevall A. Through the wall: extracellular vesicles in Gram-positive bacteria, mycobacteria and fungi. Nat Rev Microbiol. (2015) 13:620–30. 10.1038/nrmicro3480 26324094 PMC4860279

[11] Rodrigues ML Nimrichter L Oliveira DL Frases S Miranda K Zaragoza O. Vesicular polysaccharide export in *Cryptococcus neoformans* is a eukaryotic solution to the problem of fungal trans-cell wall transport. Eukaryotic Cell (2007) 6:48–59. 10.1128/EC.00318-06 17114598 PMC1800364

[12] Albuquerque PC Nakayasu ES Rodrigues ML Frases S Casadevall A Zancope-Oliveira RM. Vesicular transport in *Histoplasma capsulatum:* an effective mechanism for trans-cell wall transfer of proteins and lipids in ascomycetes. Cell Microbiol. (2008) 10:1695–710. 10.1111/j.1462-5822.2008.01160.x 18419773 PMC2562661

[13] Rodrigues ML Nakayasu ES Oliveira DL Nimrichter L Nosanchuk JD Almeida IC. Extracellular vesicles produced by *Cryptococcus neoformans* contain protein components associated with virulence. Eukaryotic Cell (2008) 7:58–67. 10.1128/EC.00370-07 18039940 PMC2224146

[14] Vallejo MC Matsuo AL Ganiko L Medeiros LC Miranda K Silva LS. The pathogenic fungus Paracoccidioides brasiliensis exports extracellular vesicles containing highly immunogenic alpha-Galactosyl epitopes. Eukaryot Cell (2011) 10:343–51. 10.1128/EC.00227-10 21216942 PMC3067469

[15] Joffe LS Nimrichter L Rodrigues ML Del Poeta M. Potential Roles of Fungal Extracellular Vesicles during Infection. mSphere (2016) 1:e00099-16. 10.1128/mSphere.00099-16 27390779 PMC4933989

[16] Oliveira DL Freire-De-Lima CG Nosanchuk JD Casadevall A Rodrigues ML Nimrichter L. Extracellular vesicles from *Cryptococcus neoformans* modulate macrophage functions. Infect Immun. (2010) 78:1601–9. 10.1128/IAI.01171-09 20145096 PMC2849392

[17] Vargas G Rocha JD Oliveira DL Albuquerque PC Frases S Santos SS. Compositional and immunobiological analyses of extracellular vesicles released by Candida albicans. Cell Microbiol. (2015) 17:389–407. 10.1111/cmi.12374 25287304

[18] Da Silva TA Roque-Barreira MC Casadevall A Almeida F. Extracellular vesicles from Paracoccidioides brasiliensis induced M1 polarization *in vitro*. Sci Rep. (2016) 6:35867. 10.1038/srep35867 27775058 PMC5075875

[19] Nimrichter L De Souza MM Del Poeta M Nosanchuk JD Joffe L Tavares Pde M. Extracellular vesicle-associated transitory cell wall components and their impact on the interaction of fungi with host cells. Front Microbiol. (2016) 7:1034. 10.3389/fmicb.2016.01034 27458437 PMC4937017

[20] Almeida F Wolf JM Da Silva TA Deleon-Rodriguez CM Rezende CP Pessoni AM. Galectin-3 impacts *Cryptococcus neoformans* infection through direct antifungal effects. Nat Commun. (2017) 8:1968. 10.1038/s41467-017-02126-7 29213074 PMC5719036

[21] Zamith-Miranda D Nimrichter L Rodrigues ML Nosanchuk JD. Fungal extracellular vesicles: modulating host-pathogen interactions by both the fungus and the host. Microbes Infect. (2018). 10.1016/j.micinf.2018.01.011. [Epub ahead of print]. 29471026 PMC6098986

[22] Deatherage BL Cookson BT. Membrane vesicle release in bacteria, eukaryotes, and archaea: a conserved yet underappreciated aspect of microbial life. Infect Immun. (2012) 80:1948–57. 10.1128/IAI.06014-11 22409932 PMC3370574

[23] Da Silva RP Puccia R Rodrigues ML Oliveira DL Joffe LS Cesar GV Extracellular vesicle-mediated export of fungal RNA. Sci Rep. (2015) 5:7763 10.1038/srep07763 25586039 PMC5379013

[24] Fachin AL Maffei CM Martinez-Rossi NM. *In vitro* susceptibility of Trichophyton rubrum isolates to griseofulvin and tioconazole. Induction and isolation of a resistant mutant to both antimycotic drugs Mutant of *Trichophyton rubrum* resistant to griseofulvin and tioconazole. Mycopathologia (1996) 135:141–3. 10.1007/BF00632334 9066154

[25] Cove DJ. The induction and repression of nitrate reductase in the fungus *Aspergillus nidulans*. Biochim Biophys Acta (1966) 113:51–6. 10.1016/S0926-6593(66)80120-0 5940632

[26] Marim FM Silveira TN Lima DSJr Zamboni DS. A method for generation of bone marrow-derived macrophages from cryopreserved mouse bone marrow cells. PLoS ONE (2010) 5:e15263. 10.1371/journal.pone.0015263 21179419 PMC3003694

[27] Rocha MC De Godoy KF Bannitz-Fernandes R Fabri J Barbosa MMF De Castro PA. Analyses of the three 1-Cys Peroxiredoxins from Aspergillus fumigatus reveal that cytosolic Prx1 is central to H2O2 metabolism and virulence. Sci Rep. (2018) 8:12314. 10.1038/s41598-018-30108-2 30120327 PMC6098058

[28] Green LC Wagner DA Glogowski J Skipper PL Wishnok JS Tannenbaum SR Analysis of nitrate, nitrite, and [15N]nitrate in biological fluids. Anal Biochem. (1982) 126:131–8. 10.1016/0003-2697(82)90118-X 7181105

[29] Oda LM Kubelka CF Alviano CS Travassos LR. Ingestion of yeast forms of Sporothrix schenckii by mouse peritoneal macrophages. Infect Immun. (1983) 39:497–504. 6832808 10.1128/iai.39.2.497-504.1983 PMC347978

[30] Garcia-Madrid LA Huizar-Lopez MD Flores-Romo L Islas-Rodriguez AE Trichophyton rubrum manipulates the innate immune functions of human keratinocytes. Centr Eur J Biol. (2011) 6:902–10. 10.2478/s11535-011-0060-6

[31] Bielska E Sisquella MA Aldeieg M Birch C O'donoghue EJ May RC. Pathogen-derived extracellular vesicles mediate virulence in the fatal human pathogen *Cryptococcus gattii*. Nat Commun. (2018) 9:1556. 10.1038/s41467-018-03991-6 29674675 PMC5908794

[32] Salazar F Brown GD Antifungal innate immunity: a perspective from the Last 10 Years. J Innate Immun. (2018) 16:1–25. 10.1159/000488539 PMC6784043 29768268

[33] Oliveira CB Vasconcellos C Sakai-Valente NY Sotto MN Luiz FG Belda Junior W. Toll-like receptors (TLR) 2 and 4 expression of keratinocytes from patients with localized and disseminated dermatophytosis. Rev Inst Med Trop Sao Paulo (2015) 57:57–61. 10.1590/S0036-46652015000100008 25651327 PMC4325524

[34] Martinez-Rossi NM Bitencourt TA Peres NTA Lang EAS Gomes EV Rossi A. Dermatophyte resistance to antifungal drugs: mechanisms and prospectus. Front Microbiol. (2018) 9:1108. 10.3389/fmicb.2018.01108 29896175 PMC5986900

[35] Yoshikawa FS Yabe R Iwakura Y De Almeida SR Saijo S. Dectin-1 and Dectin-2 promote control of the fungal pathogen Trichophyton rubrum independently of IL-17 and adaptive immunity in experimental deep dermatophytosis. Innate Immun. (2016) 22:316–24. 10.1177/1753425916645392 27189427

[36] Yoshikawa FS Ferreira LG De Almeida SR. IL-1 signaling inhibits Trichophyton rubrum conidia development and modulates the IL-17 response *in vivo*. Virulence (2015) 6:449–57. 10.1080/21505594.2015.1020274 25950847 PMC4601166

[37] Huang XZ Liang PP Ma H Yi JL Yin SC Chen ZR. Effect of culture supernatant derived from trichophyton rubrum grown in the nail medium on the innate immunity-related molecules of HaCaT. Chin Med J. (2015) 128:3094–100. 10.4103/0366-6999.169106 26608992 PMC4795267

